# Avoiding Virtual Obstacles During Treadmill Gait in Parkinson’s Disease

**DOI:** 10.3389/fnagi.2019.00076

**Published:** 2019-04-09

**Authors:** Chiahao Lu, Emily Twedell, Reem Elbasher, Michael McCabe, Colum D. MacKinnon, Scott E. Cooper

**Affiliations:** Department of Neurology, University of Minnesota, Minneapolis, MN, United States

**Keywords:** obstacle avoidance, Parkinson’s disease, adaptive gait, treadmill, postural control

## Abstract

Falls often occur due to spontaneous loss of balance, but tripping over an obstacle during gait is also a frequent cause of falls (Sheldon, 1960; Stolze et al., 2004). Obstacle avoidance requires that appropriate modifications of the ongoing cyclical movement be initiated and completed in time. We evaluated the available response time to avoid a virtual obstacle in 26 Parkinson’s disease (PD) patients (in the off-medication state) and 26 controls (18 elderly and 8 young), using a virtual obstacle avoidance task during visually cued treadmill walking. To maintain a stable baseline of stride length and visual attention, participants stepped on virtual “stepping stones” projected onto a treadmill belt. Treadmill speed and stepping stone spacing were matched to overground walking (speed and stride length) for each individual. Unpredictably, a stepping stone changed color, indicating that it was an obstacle. Participants were instructed to try to step short to avoid the obstacle. By using an obstacle that appeared at a precise instant, this task probed the time interval required for processing new information and implementing gait cycle modifications. Probability of successful avoidance of an obstacle was strongly associated with the time of obstacle appearance, with earlier-appearing obstacles being more easily avoided. Age was positively correlated (*p* < 0.001) with the time required to successfully avoid obstacles. Nonetheless, the PD group required significantly more time than controls (*p* = 0.001) to achieve equivalent obstacle-avoidance success rates after accounting for the effect of age. Slowing of gait adaptability could contribute to high fall risk in elderly and PD. Possible mechanisms may include disturbances in motor planning, movement execution, or disordered response inhibition.

## Introduction

Parkinson’s disease (PD) is a neurodegenerative disease that leads to a progressive decline in motor function, including symptoms of rigidity, tremor, bradykinesia, and postural instability and gait disorder. The gait disorder is one of the most disabling motor symptoms of PD, and at the same time, one of the most refractory to treatment, such as pharmacological (e.g., levodopa) and neuromodulatory (e.g., deep brain stimulation) treatments. One debilitating consequence of postural instability and gait disorders is increased fall risk. Over 60 percent of people with PD reported at least one fall and 70 percent of these people experienced 2 falls within a year (Wood et al., 2002). The prevalence of falls also significantly increases with disease progression (Hiorth et al., 2014).

Falling can often occur when individuals with PD fail to negotiate an obstacle during walking (Imms et al., 1977; Campbell et al., 1990; Chen et al., 1996; Defer et al., 1999; Galna et al., 2009; Brown et al., 2010). To prevent this from happening, one needs to inhibit the preplanned step and modify the ongoing cyclical movement to avoid the obstacle, i.e., a form of gait adaptation. Previous research on obstacle avoidance has indicated that movement performance was worse in people with PD compared to healthy individuals. This includes slower crossing speed (Brown et al., 2010), shorter stride length, greater stride and stance phase duration (Stegemöller et al., 2012; Vitório et al., 2010), and a larger step width (Galna et al., 2013b) and an increased number of steps when the obstacle appeared (Caetano et al., 2018). In addition, these gait impairments appear to be associated with the severity of the overall motor symptoms (Michel et al., 2009; Stegemöller et al., 2012; Galna et al., 2013b; Caetano et al., 2018).

While the spatial characteristics of movements to avoidance obstacles have been widely studied, it is also important to understand how the temporal presentation of an obstacle affects the performance. Previous literature in healthy adults has indicated that the timing of obstacle presentation can have a substantial impact on success rate for obstacle avoidance in both young and older healthy adults (Chen et al., 1994, 1996). Their results show that the success rate increases with the available response time relative to obstacle appearance. To our knowledge, no study has investigated how timing of obstacle appearance in people with PD affects their performance with respect to the available response time.

We examined obstacle avoidance in relation to available response time in people with PD compared to healthy controls during treadmill walking. In order to evaluate the performance of avoiding obstacles independent of difficulty keeping up with the speed of the treadmill, we measured each individual’s preferred overground walking speed, and examined obstacle avoidance at that speed. We hypothesized that: (1) people with PD would require more response time for obstacle negotiation compared with healthy controls, and (2) the required response time would be associated with overall disease severity in people with PD.

## Materials and Methods

### Participants

Fifty-two participants (demographics in Table 1) were included in the study: 26 people with PD and 26 healthy controls. All patients included in the study had idiopathic PD (Hoehn and Yahr scale of II–IV) (Hoehn and Yahr, 1967). Participants with PD were tested in the morning after 12-h withdrawal from anti-parkinson’s medications (practically defined off-medication state) (Defer et al., 1999). Eight of the PD participants had implanted deep brain stimulators. In these individuals, stimulation was turned off at least 1-h before the testing. Exclusion criteria included medical conditions impairing gait except for PD and diagnosis of dementia or a score of <25 on the Mini-Mental State Exam (Folstein et al., 1975). All participants gave informed consent and the protocol was approved by the University of Minnesota Institutional Review Board.

**Table 1:** Summary of participants demographics.

	Group		
	PD	Control	Control _(Age__>__45)_
Sex (Male/Female)	15/11	13/13	8/10
Age (years)^∗^	64.7 ± 9.5	52.8 ± 19.4	64.2 ± 9.1
Disease duration (years)^∗^	8.6 ± 5.9	N/A	N/A
MDS-UPDRS motor score^∗#$^	34.3 ± 17.5	N/A	N/A
MDS-UPDRS motor axial subscore^∗#∧^	8.3 ± 4.2	N/A	N/A
Overground walking speed (m/s)^∗^	1.05 ± 0.23	1.25 ± 0.16^@^	1.25 ± 0.16^@^
Overground step length (m)^∗^	0.56 ± 0.11	0.68 ± 0.06	0.67 ± 0.07
**DBS (Yes/No)**	8/18	N/A	N/A
Side (Left/Right/Bilateral)	1/2/5	N/A	N/A
Target (STN/GPi)	7/1	N/A	N/A

*^∗^Values are presented as mean ± SD. ^#^MDS-UPDRS stands for Movement Disorder Society-Sponsored Unified Parkinson’s Disease Rating Scale Revision. ^$^Two participants were excluded due to missing scored items. ^∧^One participant was excluded due to missing scored axial items. ^@^Not an error: mean ± SD walking speed was the same for Control as for Control (age > 45). N/A, not applicable; DBS, deep brain stimulation; STN, subthalamic nucleus; GPi, globus pallidus internus.*

### Protocol

Participants initially walked overground at a self-paced speed and at least 30 valid steps were acquired on an instrumented mat (Gaitrite, CIR systems, Franklin, NJ, United States) [see Galna et al. (2013a) for details on the overground walking protocol]. Average walking speed and step length were calculated and exported immediately after the completion of overground walking. Next, a 14-min practice walking session was conducted to allow participants to familiarize themselves with the treadmill (C-Mill, Motekforce Link, Culemburg, Netherlands), including 5 2-min trials at the participant’s previously measured overground walking speed, followed by 2 2-min trials at 15% faster and at 15% slower than the overground speed in random order. All practice trials were tested without projection of virtual objects. Participants were asked to wear their usual comfortable walking shoes during the overground and all treadmill walking sessions.

**FIGURE 1:**
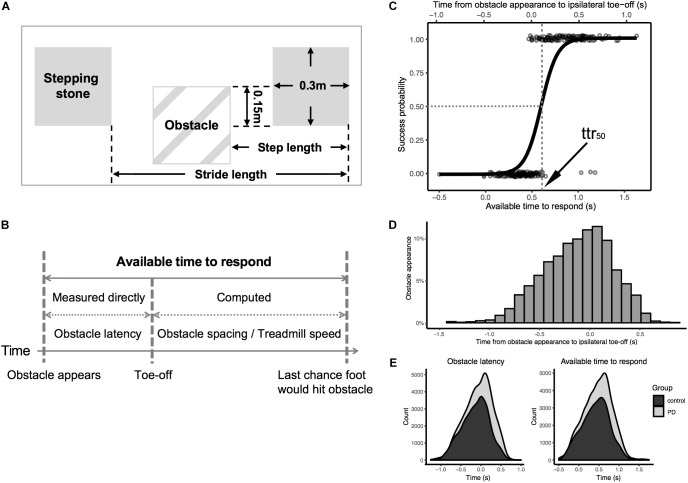
**(A)** Dimensions of virtual obstacles and stepping stones; **(B)** The distribution of timing of obstacle appearance relative to previous ipsilateral toe-off (in all participants); **(C)** Illustration for calculation of available time to respond; **(D)** Logistic regression of success/failure on available time to respond (bottom *x*-axis) and obstacle latency (top *x*-axis) in one representative participant; **(E)** density plot of obstacle latency (left)/available time to respond (right) for total number of obstacles presented to all participants separated by group.

Finally, the participants performed the obstacle avoidance task on the treadmill (0.7 m × 2.5 m) at their overground gait speed. During this task, stepping stones (blue squares, size: 0.3 m × 0.3 m), were projected onto the treadmill belt, corresponding to left and right footsteps (0.15 m left-right distance between the center of the squares), and spaced according to the individual’s average step length measured during overground gait. Movement of the stepping stones matched the speed of the treadmill belt, as if painted onto it, and participants were instructed to step on each stepping stone with the corresponding foot. Randomly and unpredictably, a stepping stone would change color (from blue to a red-white striped square) indicating that it was now an obstacle to be avoided. Participants were instructed to “step short” to avoid the obstacle (Figure 1A). Despite these instructions, they occasionally avoided an obstacle by stepping *long* (“overstep”) [overstep may be a preferred strategy for elderly (Weerdesteyn et al., 2005a,b)]. When overstep happened, they were reminded of the instructions.

The timing of obstacle appearance was generated by an algorithm of the treadmill software provided by the manufacturer. This algorithm monitored real-time heel-strike and toe-off events detected from the center of pressure (measured by the force plate built into the treadmill). From this data, it predicted the time of occurrence of the next footfall and presented an obstacle prior to the next footfall (one step before) or the one after the next foot fall (two steps before) on a random subset of gait cycles. Fortuitously, the software generated random variance in the timing of obstacles, and we exploited this feature in the present protocol. Since the variance was not under our direct control we simply measured the actual obstacle latencies relative to toe-offs. The timing of obstacle appearance relative to the previous ipsilateral toe-off was a bell-shaped distribution with 95% range from -1.0 to 0.5 s (Figure 1B). In addition, the distribution of both obstacle latency and available time to respond were similar between groups (Figure 1E, see Supplementary Material for more details).

Each participant performed six total trials. In order to reduce the predictability of obstacles, the proportion of stepping stones which changed to obstacles was set to either 1/9 on 3 of the trials or 1/6 on the other 3 trials, with the ordering varied randomly. The trial duration was 2 min for the 1/6 condition and 3 min for the 1/9 condition, which equalized the number of obstacles across trials. A safety harness was worn by all participants during treadmill walking. The harness was attached to an overhead sliding track which afforded protection against falls, without restricting the participant’s motion. Seated rest periods (2–3 min) were offered after each trial or upon participant’s request. For three participants who could not perform the obstacle avoidance task with 100% overground walking speed, the treadmill speed was reduced. For two participants it was reduced to 85%. In one of these participants, the stepping-stone spacing was modified accordingly based on step lengths measured during the 85% speed practice treadmill walking, whereas the other participant was tested at 85% speed but with the stepping-stone spacing from 100% overground walking. For the third participant, who could not perform obstacle avoidance at 100% speed, the treadmill speed was reduced to 72% and stepping-stone spacing set to the step length measured during the 85% speed practice treadmill walking trial (see Supplementary Material for more details).

Task performance was captured using a digital video camera (HDR-CX700, Sony, Tokyo, Japan) positioned in front of the treadmill to capture the treadmill belt and the lower extremities of the participant. Video was recorded at 60 fields per second with a spatial resolution of 720p.

### Data Analysis

The performance of obstacle avoidance was scored from the video recordings. In order to avoid bias in video processing, a second person, blinded to participant status (PD vs. Control) reprocessed the video data (see Supplementary Material for agreement analysis between two raters). Obstacle avoidance was classified as a success when the participant avoided stepping on the obstacle, otherwise it was classified as a failure. Each success was further categorized by two avoidance strategies, i.e., stepping short of the obstacles (as instructed: “short-step”) and stepping beyond (“overstep”). The obstacle latency was measured from the video (60 Hz, i.e., 1/60th second resolution) and defined as the time of obstacle appearance relative to ipsilateral toe-off (specifically, to the start of the swing phase which was terminated either successfully or unsuccessfully by either avoiding or stepping on the obstacle). The difficulty of avoiding the obstacle with a given obstacle latency will increase with increasing treadmill speed and decrease with increasing distance between stepping stones. For example, if an obstacle with the same obstacle latency and spacing was presented to two participants walking at different speeds, it would be more difficult for the participant with the faster walking speed to avoid the obstacle. On the other hand, if the obstacle was presented with the same obstacle latency and walking speed to two participants, but the obstacle spacing was different, it would be more difficult for the participant with the smaller obstacle spacing to avoid the obstacle. To account for this confound, we computed available time to respond (Figure 1C) with the following equation:

ART=OL+SS/TS

where *ART*, available time to respond (s); *OL*, obstacle latency to toe-off (s); *SS*, steppingstone spacing (m); *TS*, treadmill speed (m/s).

We repeated also our analysis using the raw obstacle latency data without this adjustment (see Supplementary Figure S2). This did not affect the results.

This is the time interval from obstacle appearance until the ipsilateral foot would hit the obstacle based on an average, unmodified gait cycle (Chen et al., 1994; Weerdesteyn et al., 2005a; Brown et al., 2006; Maidan et al., 2018). The primary dependent variable was defined as the time to respond at which the probability of success was 50% (ttr_50_). The ttr_50_ was computed for each participant by logistic regression of success/failure on time to respond (Figure 1D). To ensure reliability, the *p*-value of the logistic regression for each participant was verified to be less than 0.00001. The percentage of each type of success (short-step, i.e., as instructed, vs. overstep, i.e., contrary to instructions) was also calculated.

### Statistical Analysis

The primary analysis was a comparison of ttr_50_ between PD and Control groups. Because ttr_50_ varied with age (lower for younger participants) and age differed between PD and Control groups (due to the inclusion of a group of 8 Control participants less than 45 years of age), the comparison was an analysis of covariance (ANCOVA) with factor of group (PD vs. control) and age as covariate. For additional confirmation, an independent *t*-test on ttr_50_ was conducted, excluding the group of young Control participants.

The same ANCOVA and independent *t*-test or Mann–Whitney test (for skewed distribution observed in overstep success rates) were also applied to success rates as the secondary analysis. Finally, in the PD group, simple linear regression analysis was used to examine the relationship between ttr_50_ and predictors [disease duration and motor scores of Movement Disorder Society-Sponsored Unified Parkinson’s Disease Rating Scale Revision (MDS-UPDRS)], respectively. Two-tailed *p*-value threshold was set at 0.05.

## Results

A summary of the demographics of the participants is listed in Table 1 by group. The average age was significantly greater in the PD group than the controls [*t*_(50)_ = -2.7, *p* = 0.01] due to the inclusion of the eight young Controls and one young PD. The average age was not significantly different between groups when we excluded participants younger than 45 years old [*t*_(41)_ = -0.3, *p* = 0.75]. There was no difference in the proportions of male vs. female participants between the PD and control groups [all age, *X*^2^_(1,52)_ = 0.08, *p* = 0.78; age > 45, *X*^2^_(1,42)_ = 0.31, *p* = 0.58].

A small fraction of obstacle events was excluded from the analysis (0.5% in Control and 2.7% in PD) due to: footfalls not aligned with stepping stones on the gait cycle preceding the obstacle, when the participant avoided the obstacle by stepping to one side of it, or by keeping the ankle dorsiflexed so as to bear weight only on the heel. The performance of obstacle avoidance by each group is listed in Table 2. About 4 out of 5 successes were classified as stepping short (as instructed) in both control (mean ± SD, 77.6 ± 16.7%) and PD (80.6 ± 13.2%) groups.

**Table 2:** Summary of performance in obstacle avoidance (mean ± SD).

	Group		
	PD	Control	Control _(Age__>__45)_
ttr_50_ (s)	0.72 ± 0.14	0.59 ± 0.06	0.62 ± 0.04
**Success (%)**			
Total	47.0 ± 19.3	50.2 ± 14.3	46.1 ± 11.0
Overstep	7.4 ± 6.0	9.0 ± 8.5	11.1 ± 9.0
Short-step	39.4 ± 18.9	39.9 ± 15.1	34.3 ± 10.2

*ttr_50_, the time to respond at which the probability of success was 50%.*

**FIGURE 2:**
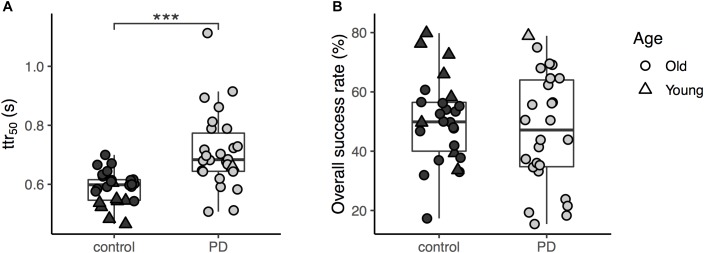
**(A)** The available time to respond (ttr_50_) of the two groups (the three asterisks shows significant difference between the groups with *p* < 0.001); **(B)** The overall success rates for obstacle avoidance task of the two groups.

**FIGURE 3:**
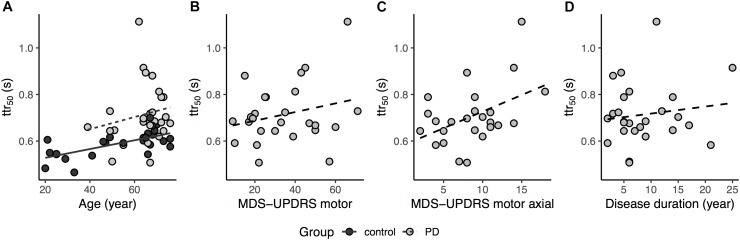
**(A)** Relationship between ttr_50_ and age for both groups, for regression lines, dashed trace represents Parkinson’s disease (PD) and solid trace Control, while light gray circle and dark gray circle represents PD and Control, respectively (age covariate, *p* < 0.001); **(B)** Relationship between ttr_50_ and MDS-UPDRS total motor scores for the PD group (two participant excluded due to missing items in MDS-UPDRS motor, dashed trace represents the regression line, *p* = 0.16); **(C)** Relationship between ttr_50_ and MDS-UPDRS motor axial subscores for the PD group, dashed trace represents the regression line (one participant excluded due to missing axial item, *p* = 0.02); **(D)** Relationship between ttr_50_ and disease duration for the PD group, dashed trace represents the regression line (*p* = 0.77).

The average ttr_50_ value was approximately 1.2 times greater in the PD group compared to healthy controls (Figure 2A). A simple between-group comparison was significant [*t*_(50)_ = -4.4, *p* < 0.001]. The ANCOVA controlling for age confirmed the results showing a significant main effect of group in the ttr_50_ [*F*_(1,49)_ = 12.2, *p* = 0.001]. The age covariate was also significant [*F*_(1,49)_ = 13.4, *p* < 0.001, Figure 3A]. For further confirmation, a simple between-group comparison, excluding participants younger than 45 years, was also significant [*t*_(41)_ = -3.1, *p* = 0.003]. Results were the same when we repeated this analysis using raw obstacle latency rather than available time to respond (see Supplementary Figure S2).

In the secondary analysis, success rates were higher in controls than in PD participants (Table 2), but this difference did not reach significance in ANCOVA [*F*_(1,49)_ = 0.16, *p* = 0.69; *t*-test, *t*_(41)_ = 0.06, *p* = 0.95, Figure 2B] though the age covariate was significant [*F*_(1,49)_ = 9.7, *p* = 0.003]. We also found no significant group effect for short-step success rates [*t*_(41)_ = -0.78, *p* = 0.44] in participants older than 45 years of age. For overstep success rate, the between-group difference in participants older than 45 years of age was also not significant (*U* = 274, *p* = 0.24).

Linear regression analysis in the PD group showed MDS-UPDRS motor score [*F*_(1,22)_ = 2.08, *p* = 0.08, *R^2^* = 0.08, two participants excluded due to incomplete scores] was not significantly related to the ttr_50_ although there was a non-significant trend to increasing ttr_50_ with increasing motor scores in MDS-UPDRS (Figure 3B). However, the MDS-UPDRS motor axial subscore [Factor 1 items in Goetz et al. (2008)] was significantly related to ttr_50_ [*F*_(1,23)_ = 5.14 (missing data in one participant), *p* = 0.03, *R^2^* = 0.15, Figure 3C]. There was no significant relationship between ttr_50_ and disease duration [*F*_(1,24)_ = 0.58, *p* = 0.45, *R^2^* = 0.02] although there was a non-significant trend to increasing ttr_50_ with increasing disease duration (Figure 3D). There was no relation of success rate to MDS-UPDRS motor scores [*F*_(1,22)_ = 0.39, *p* = 0.54, *R^2^* = 0.03], MDS-UPDRS motor axial subscores [*F*_(1,23)_ = 2.07, *p* = 0.16, *R^2^* = 0.04] and disease duration [*F*_(1,24)_ = 0.07, *p* = 0.79, *R^2^* = 0.04]. In addition, there was no difference in ttr_50_ between PD patients with vs. without deep brain stimulator [DBS, *t*_(24)_ = -1.91, *p* = 0.07] (All DBS participants tested OFF-stim, with at least 1-h washout) and in ttr_50_ between more vs. less affected legs in PD patients [paired *t*-test, *t*_(24)_ = 0.02, *p* = 0.98].

## Discussion

To our knowledge, this is the first study to assess how PD affects the capacity for obstacle avoidance as a function of the available time to respond. Our results showed that the probability of successfully avoiding an unexpected obstacle depended strongly on the available time to respond in both controls and people with PD. In addition, we found that PD participants required more time than controls to achieve an equivalent probability of avoiding the obstacle.

Our control participants replicate the finding by Potocanac et al. (2014), who showed a similar dependence on time-to-respond in a smaller sample of neurologically healthy participants (Potocanac et al., 2014). In addition to being larger, our sample of control participants also spanned a wider age range and included elderly participants. We also varied time-to-respond with higher resolution, i.e., continuously, rather than in discrete increments. The logistic regression curve relating the probability of successful avoidance to available time was shifted to the right in older as compared to younger Controls (i.e., older participants required more time than younger ones to achieve an equivalent probability of avoiding the obstacle).

The logistic regression was also shifted to the right in the PD group relative to Controls (i.e., PD participants required more time than Controls to achieve an equivalent probability of avoiding the obstacle) even after accounting for the effect of age. Thus, consistent with our hypothesis, our results demonstrate that people with PD required more time to respond (greater ttr_50_) in order to avoid obstacles during treadmill walking compared to healthy adults. Although ttr_50_ increased with increasing disease duration and with increasing MDS-UPDRS total motor score, neither relation reached significance. However, ttr_50_ was significantly related to MDS-UPDRS axial subscores. This suggests that prolongation of ttr_50_ is predominantly associated with postural and gait dysfunction, and thus, may be a distinct domain of impairment rather than more generalized measures of disease severity.

Previous obstacle avoidance studies in people with PD used a treadmill and three-dimensional obstacles (van Hedel et al., 2006; Michel et al., 2009; Snijders et al., 2010; Nanhoe-Mahabier et al., 2012; Stegemöller et al., 2012). With three-dimensional obstacles the larger limb movement required to step over the obstacle may be additionally difficult for Parkinsonian patients due to hypometria, a spatial deficit, thereby confounding spatial and temporal impairment. In contrast, we used two-dimensional virtual obstacles with instructions to shorten the step to avoid the obstacle. This reduction in the spatial demands of the task allowed a more direct comparison of the temporal aspect of obstacle avoidance between Control and PD. In addition, although we expected the short-step instructions would provoke episodes of freezing of gait (FOG) (Chee et al., 2009), such episodes were actually rare, even in patients for whom FOG was a prominent symptom. This might simply reflect the irregular and unpredictable nature of FOG which makes it hard to provoke reproducibly in a laboratory setting, or the facilitatory effect of the visual cues (“stepping stones”) in our experiments.

Shorter steps (in PD) and faster gait (in controls) reduce the available time to respond to an obstacle, for a given obstacle latency, which would be expected to reduce success probability. However, this cannot explain the difference we saw between PD and control subjects, because we analyzed success probability as a function of time-to-respond, rather than of the obstacle latency. Therefore, the prolonged response time in our PD participants cannot be completely explained by walking speed or step length.

The obstacle avoidance task used in the current study is similar to a classical response inhibition task (Go-NoGo or Stop Signal Task). People with PD do exhibit impaired response inhibition (Gauggel et al., 2004; Dirnberger and Jahanshahi, 2013). In our task, participants were asked to alter their stride to avoid an obstacle. This differs from a simple response inhibition task as it may require not only inhibiting the default “prepotent” response, but also replacing it with a different one, much like a Stroop test (Stroop, 1935). Furthermore, we observed *three* different behaviors in response to the obstacle appearance. Not only did we see the prepotent (stepping on the obstacle) and the instructed response (stepping short of the obstacle), but subjects also demonstrated a third behavior, that is, overstepping so that the foot landed on the far side of the obstacle. Overstepping was deployed appropriately to achieve obstacle avoidance, but was contrary to instructions. This appears to represent a preferred obstacle-avoidance response. The fact that overstepping occurred preferentially for obstacles with shorter time to respond (see Supplementary Material) suggests that this strategy is another prepotent response which must be inhibited in order to comply with task instructions. Another potential explanation for overstepping could be that lengthening the step would increase the anterior-posterior base of support, thus providing increased stability in the sagittal plane. Response inhibition is often studied in highly artificial tasks. The present study may shed light on how response inhibition modulates a real-world, and functionally very important daily activity, namely gait.

Functional neuroimaging studies have provided evidence that the prefrontal cortex plays an important role in response inhibition or response switching. Maidan et al. (2016) showed a significant increase in prefrontal activation during obstacle negotiation compared to normal walking in people with PD, but this increase was not seen in aged matched healthy adults (Maidan et al., 2016). This is in keeping with the growing body of evidence relating prefrontal cortical attentional and executive function to gait (Herman et al., 2010; Lord et al., 2010; Snijders et al., 2010; Smulders et al., 2013).

Gait speed may be a proxy for PD disease stage (Paker et al., 2015), and in our PD participants, walking speed was inversely related to MDS-UPDRS motor scores. A factor that may have contributed to reduced walking speed and shortened step length during treadmill walking in the PD group is visual information load. Participants with PD would perceive more stepping stones given the same projection area on the treadmill belt compared to control participants who walked with greater speed and step length. Thus, this additional load of visual information may exacerbate the performance deficit of these participants, which may, in part, contribute to the prolonged time to respond.

Success rates were higher in controls than in PD participants, but this difference did not reach significance. Relatively high success rates in PD patients may be a characteristic of externally cued tasks (van Hedel et al., 2006) or occur when subjects engage a subcortical “fast adjustment network” (Snijders et al., 2010; Potocanac and Duysens, 2017). We also did not find a significant relationship between success rate and the MDS-UPDRS motor scores (PD only). This is expected because success rate depends on obstacle latency. To compare success rates, time-to-respond should be matched, or alternatively, as in the present paper, one can directly compare the function relating success probability to time-to-respond.

In the current study, obstacle avoidance occurred in the context of a baseline walking task in which participants were asked to step on virtual stepping stones. The reason for this instruction was to maintain a stable baseline of stride length and visual attention, so that the step preceding an obstacle was similar for every obstacle. However, our use of stepping stones could have affected our results in several ways. First, although we attempted to set the spacing of the stones to match the individual participant’s overground step length, the average of left and right step lengths was used. Since most PD participants had asymmetrical step lengths, this may have normalized the asymmetry of their treadmill gait. Future studies may investigate how gait asymmetry affects obstacle avoidance in PD since it has been shown that elderly with high fall risk may demonstrate more asymmetry in lower limbs when stepping over obstacles compared to young control and elderly with low fall risk (Di Fabio et al., 2004). Second, it is well known that providing a visual cue improves gait in PD (Lim et al., 2005). Third, the requirement to step on the stepping stone may have constituted an additional cognitive load on participants, on which the obstacle-avoidance requirement was superimposed. Therefore, a potential explanation of the increase of the available time to respond in the PD group could be the cognitive cost of dual task, which involved processing of both stepping stones and obstacles. Yet, previous research has shown that simultaneous external cueing did not affect obstacle crossing performance in PD groups with and without FOG during treadmill walking (Nanhoe-Mahabier et al., 2012) (although in that study, the cues were auditory rather than visual).

It is worth mentioning that the average available response time of our neurologically healthy older controls in obstacle crossing was higher (0.6 s) than in a previous study (0.2–0.35 s) (Weerdesteyn et al., 2005a) for similar success rates. The difference could be that the average treadmill speed was substantially slower in the Weerdesteyn et al. (2005a) study compared to the current study (0.8 vs. 1.2 m/s). In addition, their obstacle always dropped at the same location and the sound of the obstacle landing may have acted as a supplemental acoustic cue. Chen et al. (1994) reported 50% success at 280 ms available response time in healthy elderly participants when using a virtual obstacle during overground gait. The constraint on gait speed regulation imposed by the use of a treadmill in our study may have increased the difficulty of our task through a dual-task effect and thus increased the response times. Other implementation details (e.g., visual salience of the obstacles) may also have played a role.

Our study has limitations, and the findings should be interpreted with caution. First, gait could differ between overground and treadmill walking (Bello et al., 2014; Malatesta et al., 2017), although the literature on this topic is equivocal (e.g., Frenkel-Toledo et al., 2005; Riley et al., 2007; Hollman et al., 2016) and may depend on the specific population (e.g., Watt et al., 2010) and on laboratory-specific details. Second, it is possible that the gait speed and step length which were natural for overground walking were less natural on the treadmill. In that case, our results may pertain more to more challenging gait. The gait speed we chose was intended to approximate “natural,” i.e., overground gait, subject to the constraint that the experiment required a treadmill. Overground gait seemed more relevant to community ambulation, activities of daily living and fall risk, etc., Although we instructed participants to step short of the obstacle, yet overstepping sometimes occurred. Thus, participants had two strategies that could be implemented, but were instructed to preferentially select one of these (step short). The process of selection and suppression of strategies may have increased response time since this could impose an additional cognitive load analogous to dual tasking, which is known to affect gait in PD (Bond and Morris, 2000; Yogev et al., 2005; Rochester et al., 2008; Amboni et al., 2013) (see Supplementary Material for a more detailed analysis of short-stepping/overstepping rates). Future studies are warranted to investigate how the adopted avoidance strategy is influenced by the available time to respond *without* the constraint of specific instructions. In addition, we were unable to extrapolate our findings to potential fall risk due to lack of comprehensive falls history of our sample population. In order to avoid statistical confounding from systematic differences in step length and gait speed between PD and control groups, we analyzed our data in terms of available time to respond, based on obstacle latency adjusted for gait speed and step length. A limitation of this approach is that that one cannot disentangle effects of speed and target spacing. However, our conclusions were unaffected when data was reanalyzed in terms of raw, unadjusted obstacle latency (see Supplementary Material). Another limitation was that we were unable to assess the effect of learning or fatigue on the obstacle avoidance by comparing earlier vs. later trials, or earlier vs. later obstacles within a trial. This is because we pooled all trials in order to fit the logistic regression with the best accuracy. Therefore, we cannot exclude the possibility that the difference between PD participants and controls was that the PD participants required more practice to learn the task. Finally, the obstacle latency was estimated at 60 Hz due to the time resolution of the video.

This study shows that the available time required to respond in obstacle negotiation was prolonged in people with PD during preferred speed treadmill gait compared to neurologically healthy adults. Furthermore, the prolonged response time was associated with the severity of Parkinsonian axial motor dysfunction.

## Author Contributions

CL and SC conceived the study, analyzed the data, developed the methodology, administered the project, and wrote, reviewed, and edited the manuscript. CM conceived the study, investigated the results, developed the methodology, and wrote, reviewed, and edited the manuscript. ET, RE, and MM developed the methodology, analyzed the data, and reviewed and edited the manuscript.

## Conflict of Interest Statement

The authors declare that the research was conducted in the absence of any commercial or financial relationships that could be construed as a potential conflict of interest.
