# Supplementary material for: Avoiding Virtual Obstacles During Treadmill Gait in Parkinson’s Disease
Source: Front Aging Neurosci. 2019 Apr 9;11:76. doi: 10.3389/fnagi.2019.00076 (PMC6465417; doi:10.3389/fnagi.2019.00076)
Supplement: Supplementary file 1 [file Data_Sheet_1.docx]

Supplementary Material

The software version allowed limited control over the timing of obstacles, namely, settings of "one step to respond" and "two steps to respond." The actual timing of obstacle appearance resulted from the interaction of this setting with the real-time gait-event detection algorithm (based on center-of-pressure trajectory) of the treadmill. Consequently, we computed observed times of obstacle appearance, as measured from video recordings, rather than device settings. To achieve a broad range of obstacle timings, we did 4 trials at a setting of "one step to respond" and 2 trials at "two steps to respond" with pseudorandomized trial order across participants. The actual obstacle timing distributions in the two conditions overlapped, giving the overall bell-shaped distribution shown in Figure 1B. The manufacturer subsequently provided us with a customized version of the software that allowed more fine-tuning of obstacle timing, but this was not available for the data reported here. In addition, the following plot shows that the distribution of both obstacle latency and available time to respond were similar between groups.

**Figure 1**

Three subjects were unable to perform the obstacle-avoidance task with the treadmill set to 100% of their full overground walking speed. These subjects were tested reduced speed (two at 85% and one at 72% of their overground walking speed). We reanalyzed the data excluding those three participants and this did not affect our findings. For example, the statistical analysis for ttr_50_ remained similar. The ANCOVA controlling for age showed significant main effect of groups in the ttr_50_ (*F*_(1,46)_ = 10.3, *p* = 0.002) and the age covariate was also significant (*F*_(1,46)_ = 11.8, *p* = 0.001). The between-groups comparison was still significant (*t*_(38)_ = -2.7, *p* = 0.01).

We reconfirmed our results with “raw” obstacle latencies, uncorrected for step length or gait speed. That is, instead of ttr_50_ we computed ol_50_, the obstacle latency at which the probability of successfully avoiding the obstacle was 0.5 (50%). For the analysis with obstacle latency, the average value was approximately 2 times greater in the PD group (0.16 ± 0.10 s) compared to healthy controls (0.07 ± 0.05 s). A simple between-group comparison was still significant (*t*_(50)_ = -4.9, *p* < 0.001). The ANCOVA controlling for age showed a significant main effect of group in the obstacle latency (*F*_(1,49)_ = 14.7, *p* < 0.001). The age covariate was also significant (*F*_(1,49)_ = 29.5, *p* < 0.001). For further confirmation, a simple between-group comparison, excluding participants younger than 45 years, was also significant (*t*_(41)_ = -3.5, *p* = 0.001).

**Figure 2**


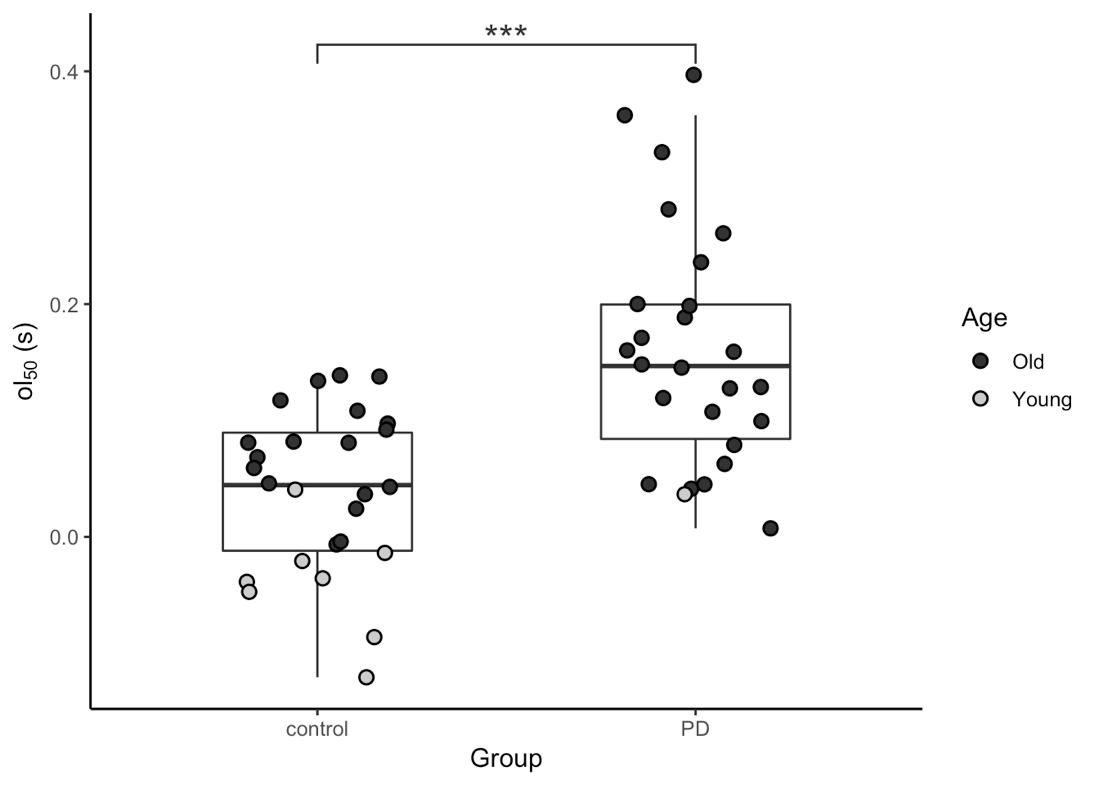


In 21 randomly selected participants (12 PD and 9 Control) the calculated available time to respond (i.e., ttr_50_) from one rater agreed extremely well with ttr_50_ as computed based on the other rater except for one PD participant whose data were therefore excluded from the analysis. The inter-rater reliability was then verified with the following two procedures, 1) examining the success rates using intraclass correlation coefficient (ICC), the results showed the 95% confidence interval for ICC is between 0.955 and 0.992 with *p* < 0.0001. 2) examining the ttr_50_ values using linear correlation, the analysis showed a strong correlation between two raters with *R*^2^ = 0.902 with *p* < 0.0001.

# Figure 3

In this study, participants were instructed to avoid the obstacle by stepping short. Stepping to the side, or stepping over the obstacle were explicitly prohibited. While side stepping was rare (only 0.2 %, possibly because the width of the obstacle left little room for this tactic) overstepping occurred on 20.2 % of obstacles successfully avoided. This was not because participants misunderstood instructions: when they overstepped we reminded them of the prohibition, and participants who overstepped often commented that they recognized the error, but were unable to avoid overstepping. Overstepping was apparently a preferred response to an obstacle. This may be because overstepping is an easier-to-execute strategy: Unlike shortstepping, overstepping actually postpones heel-strike, which allows extra time to adjust the stride.

There was considerable variation among participants in how often overstepping occurred. PD participants tended to use it less, possibly because it required an extra-large hip flexion; Parkinsonian hypokinesia presumably interfered with that. Moreover, ttr_50_ differed between shortstepping and overstepping. Although it was impossible to compute ttr_50_ for overstepping alone (most participants had too few oversteps to fit the logistic regression) we could compute ttr_50_ including both over- and short-step responses ("loose"), and compare it with ttr_50_ including only short stepping ("shortstep"). ttr_50_ was greater for the shortstep than for the loose case, from which we can infer that ttr_50_ for oversteps is shorter than for shortsteps. Moreover, as the Supplementary Figure 4 shows, this effect is greatest in participants who most frequently used the overstepping strategy.

We interpret this observation as follows: each participant had a threshold of time to respond (ttr). For obstacles which appeared later than this (i.e. especially difficult obstacles) they employed (involuntarily) the easier "cheating" overstep tactic. This threshold varied between participants. In participants with a low ttr threshold (i.e. those who "cheated less") the number of oversteps was smaller, and the effect of including oversteps in the computation of ttr_50_ was therefore smaller as well. Participants with a higher threshold (who "cheated more") had more oversteps and the effect of including oversteps was greater.

Importantly, including vs. excluding oversteps does not affect our conclusions, since the groups differed significantly in both ways. PD and control participants did not differ in the amount of overstepping (PD vs Control_age>45_: 7.4% vs 11.1%, *p* = 0.24). We argue that including oversteps is more correct. If we only include short steps, then, in essence, each participant by "choosing" to overstep, can exclude any particular obstacle from the ttr_50_ computation. If this is done preferentially for shorter ttr, then shorter ttr are excluded from the computation of ttr_50_, and ttr_50_ is artifactually elevated.

The second conclusion we draw from the use of overstepping in our experiments is that, in our task, there are actually *three* competing motor responses. Unlike a classical psychological "response inhibition" or "response switching" task (e.g. Stroop test) gait is an ethological meaningful behavior. Participants come to the laboratory with a lifetime's experience in walking, and already have a learned, or perhaps innate strategy for obstacle avoidance, which evidently competes with the consciously intended one.

**Figure 4**

# Figure Legends

**Figure 1, (A)** density plot of obstacle latency for total number of obstacles presented to all participants separated by group; **(B)** density plot of available time to respond for total number of obstacles presented to all participants separated by group.

**Figure 2,** The estimated obstacle latency at 50% success rate (ol_50_) of the two groups (the three asterisks shows significant difference between the groups with *p* < 0.001)

**Figure 3,** Estimated ttr_50_ values for the randomly selected 20 participants from rater 2 and rate 1 (dashed trace represents the regression line, (*F*_(1,18)_ = 165.4, *p* < 0.001))

**Figure 4,** Effect of overstep percentage on ttr_50_ estimated with different success definitions (triangle represents ttr_50_ estimated with shortstep only, circle represents ttr_50_ estimated with both shortstep and overstep; grey filled symbol represents PD and black filled symbol represents control)
